# Glycycoumarin exerts anti-liver cancer activity by directly targeting T-LAK cell-originated protein kinase

**DOI:** 10.18632/oncotarget.11610

**Published:** 2016-08-25

**Authors:** Xinhua Song, Shutao Yin, Enxiang Zhang, Lihong Fan, Min Ye, Yong Zhang, Hongbo Hu

**Affiliations:** ^1^ Beijing Advanced Innovation Center for Food Nutrition and Human Health, College of Food Science and Nutritional Engineering, China Agricultural University, Beijing 100083, China; ^2^ College of Veterinary Medicine, China Agricultural University, Beijing 100193, China; ^3^ The State Key Laboratory of Natural and Biomimetic Drugs, School of Pharmaceutical Sciences, Peking University, Beijing 100191, China; ^4^ The Key Laboratory of RNA Biology, Institute of Biophysics, Chinese Academy of Sciences, Beijing 100101, China

**Keywords:** licorice, glycycoumarin, anti-liver cancer, T-LAK cell-originated protein kinase, p53

## Abstract

Glycycoumarin (GCM) is a major bioactive coumarin compound isolated from licorice and the anti-cancer activity of GCM has not been scientifically addressed. In the present study, we have tested the anti-liver cancer activity of GCM using both in vitro and in vivo models and found for the first time that GCM possesses a potent activity against liver cancer evidenced by cell growth inhibition and apoptosis induction in vitro and tumor reduction in vivo. Mechanistically, GCM was able to bind to and inactivate oncogenic kinase T-LAK cell-originated protein kinase (TOPK), which in turn led to activation of p53 pathway. Our findings supported GCM as a novel active compound that contributed to the anti-cancer activity of licorice and TOPK could be an effective target for hepatocellular carcinoma (HCC) treatment.

## INTRODUCTION

Hepatocellular carcinoma (HCC) is currently the second leading cause of cancer-related death worldwide [1]. Molecular-targeted treatment for HCC with sorafenib, a multikinase inhibitor that blocks growth factor receptor-mediated signaling, demonstrated an encouraging clinical outcome and represents future trends for the treatment of HCC [2]. Novel molecular-targeted agents are being intensively investigated for the improvement in the management of HCC [3]. T-LAK cell-originated protein kinase (TOPK/PBK), a member of serine-threonine mitogen-activated protein kinase kinase family, is found highly expressed in certain types of cancer including breast [4], colon [5] and lung [6] cancer and HCC [7], and activation of TOPK is closely linked to the tumor development. It has been shown that inactivation of TOPK by its inhibitors strongly suppressed tumor growth in xenograft models of human colon [8] and lung cancer [9]. We speculated that TOPK could also be an effective target for HCC therapy, and the agents that can block TOPK could also be effective against HCC.

**Figure 1:**
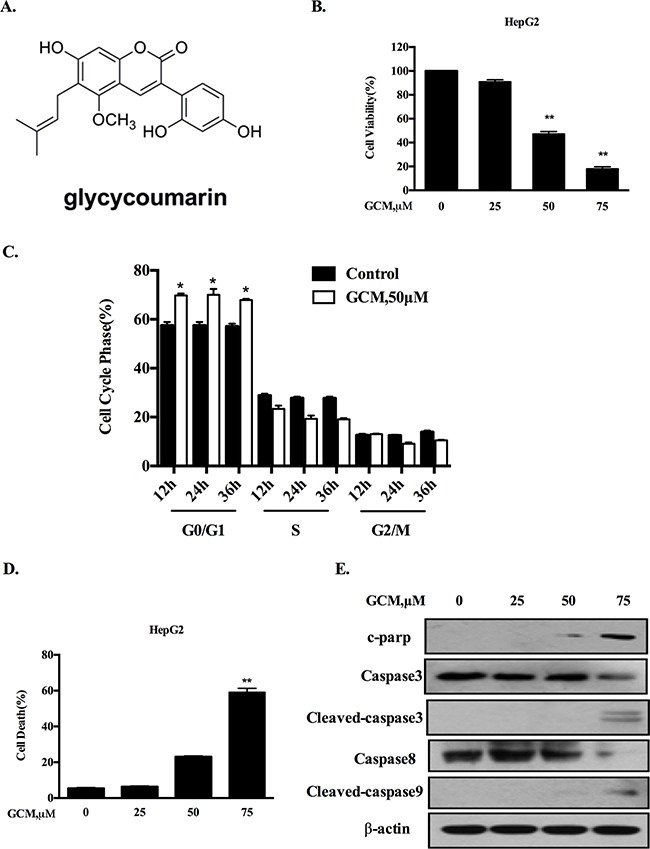
GCM induces cell cycle arrest and apoptosis in HepG2 cells **A.** Chemical structure of GCM. **B.** Overall inhibitory effects of GCM on HepG2 cells measured by crystal violet staining. **C.** Influences of GCM on cell cycle distribution measured by flow cytometry following staining with propidium iodide. **D.** Apoptosis induction in response to GCM assessed by Annexin V/FITC Staining. **E.** Activations of caspases by GCM analyzed by western blotting.

Herbal medicine has been used for centuries to manage various diseases including cancer. Licorice, one of the most popular employed medicinal plants in the Traditional Chinese Medicine, has been found to process multiple biological functions including anti-inflammatory, antivirus, anti-cancer, anti-spasmodic and hepatoprotective effects [10–12]. It has been documented that the main bioactive chemical constituents in licorice include flavonoids, triterpene saponins, and coumarins [13]. Glycycoumarin (GCM) (Figure 1A) is a representative coumarin in licorice with favorable pharmacologic feature in vivo [13]. It has been shown that GCM possesses anti-viral [14, 15], anti-inflammatory [16], anti-spasmodic [17] and liver protective effect [18]. We hypothesized that GCM could be one active component of licorice that contributed to its anti-cancer activity. In the present study, the anti-cancer activity of GCM has been evaluated using both in vitro and in vivo models. The results demonstrated that GCM is highly effective against liver cancer in both cell culture and HepG2 xenograft models. Mechanistically, the anti-cancer activity of GCM was attributed to its ability to directly inactivate TOPK, which in turn led to p53-dependent cell growth inhibition and apoptosis induction.

## RESULTS

### GCM induces cell cycle arrest and apoptosis in HepG2 cells

To evaluate the overall inhibitory effects of GCM on liver cancer cells, HepG2 hepatoma cells were exposure to various concentrations of GCM for 36 h and crystal violet staining was employed to measure the cell viabilities. As shown in Figure 1B, exposure to GCM resulted in a dose-dependent inhibitory effect on HepG2 cells. To investigate if cell cycle arrests mechanism contributed to the overall inhibitory action of GCM, the changes of cell cycle distribution in response to GCM were examined by flow cytometry. As shown in Figure 1C, exposure to 50μM of GCM caused a significant increase of G1 cells from 12 to 36 h. The results suggested that GCM inhibited cell proliferation by arresting cells at G1 phase. In addition to cell cycle arrests, morphologic observations of HepG2 cells treated with GCM suggested involvement of cell death induction in the overall inhibitory effect. We then measured cell death induction by GCM in HepG2 cells using annexin v/PI staining. As shown in Figure 1D, treatment with GCM for 36 h resulted in a concentration-dependent increase of cell death. These results were further validated by western blotting analysis of caspases and PARP, in which, GCM at high concentration caused a significantly increased cleavages of caspases and PARP (Figure 1E). These data indicated that GCM was able to induce a significant G1-phase cell cycle arrest and cell death induction in HepG2 liver cancer cells.

### Activation of p53 signaling is responsible for cell cycle arrest and apoptosis in response to GCM

**Figure 2:**
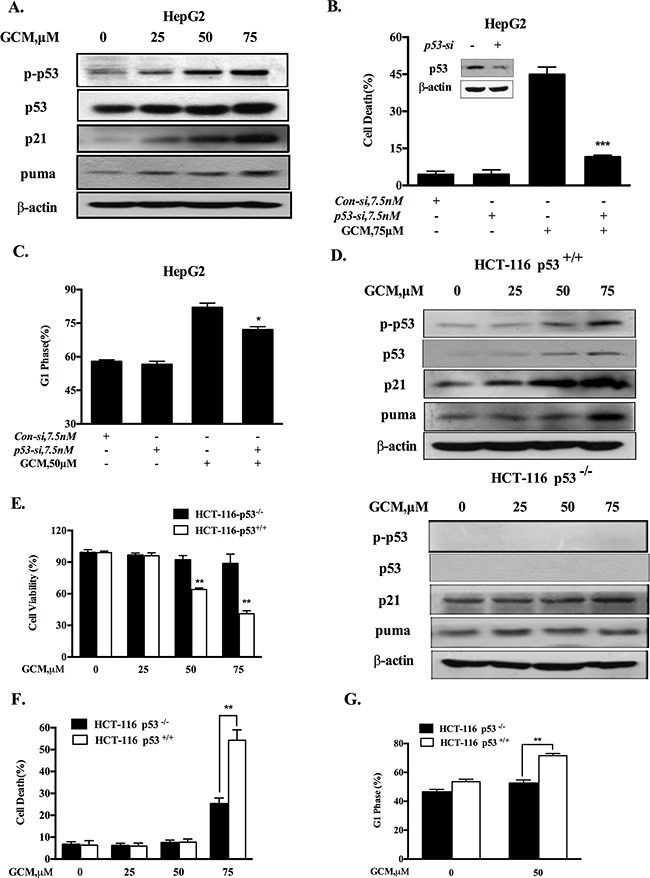
Activation of p53 signaling is responsible for cell cycle arrest and apoptosis in response to GCM **A.** GCM activated p53 signaling pathway in HepG2 cells. The cells were exposure to various concentrations of GCM for 24 h and the changes of p-p53, p21 and puma were analyzed by western blotting. **B.&C.** Effects of p53 inhibition by RNAi on GCM-induced cell death and cell cycle arrest in HepG2 cells. The cells were transfected with 7.5 nmol/L of p53 siRNA or non-targeting siRNA for 24 h and then treated with GCM for 24 h. Cell death was measured by Annexin V/FITC Staining (B) and cell cycle distribution was assessed by flow cytometry following staining with propidium iodide (C). **D.** GCM induced p53-dependent up-regulation of p21 and puma in HCT-116 cells. HCT-116 p53+/+ or p53−/− cells were treated with various concentrations of GCM for 24 h and the changes of p-p53, p21 and puma were analyzed by western blotting. **E.** Overall inhibitory effects of GCM on HCT-116 p53+/+ or p53−/− cells measured by crystal violet staining. **F.** Effects of GCM on cell cycle distribution in HCT-116 p53+/+ or p53−/− cells measured by flow cytometry following staining with propidium iodide. **G.** Apoptosis induction by GCM in HCT-116 p53+/+ or p53−/− cells analyzed by Annexin V/FITC Staining.

HepG2 cells contain wild-type p53 that is the first identified and the best known tumor suppressor through mechanisms involved in regulation of cell cycle and apoptosis. We hypothesized that G1-phase cell cycle arrest and cell death induction by GCM might be mediated by p53 signaling pathway. To test this hypothesis, we first examined whether p53 was activated in response to GCM exposure in HepG2 cells. As shown in Figure 2A, exposure to GCM caused a dose-dependent increase of p53 phosphorylation, accompanied by up-regulation of its two transcriptional targets p21 and puma. To determine the functional role of p53 activation, we tested influences of p53 inhibition by RNAi approach on GCM-induced cell death and cycle arrest in HepG2 cells. As shown in Figure 2B&2C, knockdown of p53 led to a dramatically decreased cell death induction (Figure 2B) and a significantly attenuated cell cycle arrest (Figure 2C) in response to GCM exposure. We further validated this notion in p53 WT/KO HCT-116 colon cancer cells. As shown in Figure 2D, enhanced p53 phosphorylation and p21/puma expression were observed in HCT-116 with wild-type p53. However, no such changes were detected in p53-knockout HCT-116 cells. These results suggested that p53 was transcriptional activated by GCM in colon cancer cells. We then measured the overall inhibitory effects of GCM in p53 wild-type and knockout HCT-116 cells. As shown in Figure 2E, HCT-116 cells with wild-type p53 were more sensitive to GCM than p53-knockout cells. In agreement with the overall inhibitory effect, a reduced cell death induction by GCM at 75μM was observed in p53 knockout HCT-116 cells relative to p53 wild-type cells (Figure 2F). Moreover, exposure to 50μM GCM for 24 h induced a stronger G1-phase cell cycle arrest in p53 wild-type HCT-116 cells (from 53 % to 71%) than that of p53 knockout cells (from 46% to 52%), (Figure 2G). These results indicated that p53 activation was involved in GCM-induced cell cycle arrest and cell death in cancer cells tested.

### Activation of p53 by GCM is associated with suppression of TOPK

**Figure 3:**
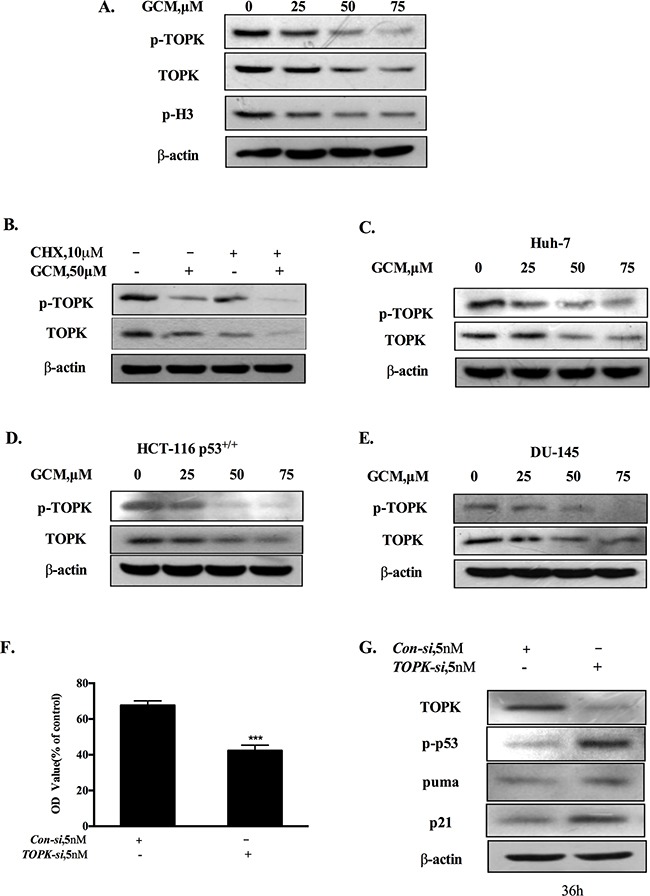
Activation of p53 by GCM is attributed to suppression of TOPK **A.** GCM inhibited TOPK in HepG2 cells. The cells were treated with various concentrations of GCM for 24 h and the changes of phospho-, total TOPK and its substrate histone H3 phosphorylation were determined by western blotting. **B.** GCM promoted TOPK degradation in HepG2 cells. The cells were treated with 50μM of GCM in the presence or absence of CHX and the expression of TOPK was analyzed by western blotting. **C.** GCM inhibited TOPK in Huh-7 cells. **D.** GCM inhibited TOPK in HCT-116 cells. **E.** GCM inhibited TOPK in DU145 cells. **F.** Overall inhibitory effects of TOPK knockdown on HepG2 cells measured by crystal violet staining. **G.** TOPK knockdown activated p53 in HepG2 cells. The cells were transfected with 5 nmol/L of TOPK siRNA or non-targeting siRNA for 36 h and the changes of p-p53, p21 and puma were analyzed by western blotting.

It has been shown that TOPK is a binding partner and negative regulator of p53 [19]. We hypothesized that inhibition of TOPK could contribute to activation of p53 in response to GCM exposure. The changes of phospho-, total TOPK and its substrate histone H3 phosphorylation in response to various concentrations of GCM in HepG2 cells were analyzed by western blotting. As shown in 3A, GCM treatments resulted in a dose-dependent decrease of phospho- and total TOPK. Accordingly, phosphorylation of its substrate histone H3 was also inhibited by GCM in a same manner. To determine whether down-regulation of total TOPK was due to its degradation, we assessed changes of TOPK protein level in response to GCM in the presence or absence of cycloheximide (CHX), a protein synthesis inhibitor. As shown in Figure 3B, when new protein synthesis was blocked, TOPK protein level was still decreased by GCM. These results suggested that TOPK was suppressed by GCM through inhibiting its phosphorylation and promoting its degradation. To examine the general application of this inhibitory effect, additional liver cancer cell line Huh-7, colon cancer cell line HCT-116 and prostate cancer cell line DU145 were employed and the results are shown in Figure 3C-3E. The results demonstrated that a similar inhibitory action on TOPK was observed in all the three cell lines tested in response to GCM. To assess the biological significance of TOPK inhibition, we measured the effects of TOPK inhibition by its specific siRNA on cell viability of HepG2 cells. As shown in Figure 3F, knockdown of TOPK resulted in a significant reduction of cell number in HepG2 cells. Consistent with the decrease of cell viability, silencing of TOPK triggered activation of p53 signaling pathway evidenced by increase of p53 phosphorylation followed by up-regulation of its two transcriptional targets p21 and puma (Figure 3G). Together, these data supported that activation of p53 was likely attributed to inhibition of TOPK in response to GCM in HepG2 cells.

### TOPK is a direct target of GCM

**Figure 4:**
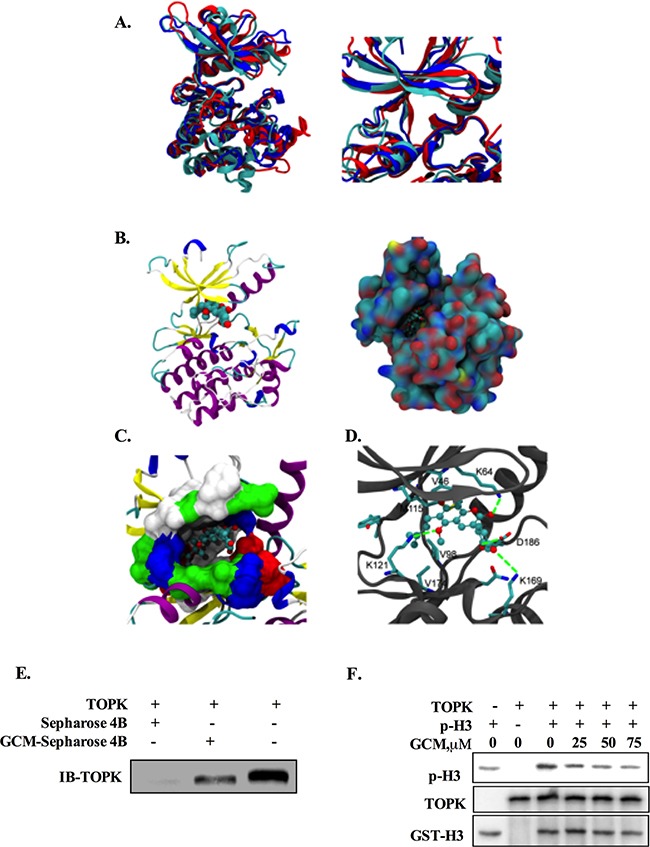
TOPK is a direct target of GCM **A.** Three predicted structures of the full-length TOPK (left) and their ATP-binding sites (right). **B.** The docking model of GCM / TOPK complex structure. GCM is shown in sphere representation (carbons are colored by cyan. TOPK is shown as a cartoon model, left) and in surface representation of TOPK (the location of GCM is shown in ball-stick, right). **C.** Binding site of TOPK with GCM. The ATP-binding site of TOPK is shown in surface representation (white: hydrophobic residues, blue: residues with positive charge, red: residues with negative charge, green: other polar residues). GCM is shown in ball-stick representation. **D.** The detailed Interaction between TOPK and GCM. Carbons on TOPK are colored grey. GCM is shown in ball-stick representation and key residues on TOPK are shown in stick representation. Carbons, oxygen, nitrogen and sulfur atoms are colored cyan, red, blue and yellow respectively. **E.** GCM binds directly to TOPK. Sepharose 4B was used for binding and pull-down assay. Lane 1, the negative control, indicating no binding between TOPK and beads alone; Lane 2, TOPK binds to GCM-Sepharose 4B beads; lane 3, input control. **F.** GCM inhibited TOPK activity in vitro in a dose dependent manner. An inactive GST-Histine H3 protein was used as the substrate with active TOPK and the p-histone H3 was measured by western blotting.

Having found the inhibitory effect of GCM on TOPK, we next asked whether TOPK was a direct target of GCM. To theoretically determine the possibility, molecular docking of GCM and TOPK complex was performed using both Autodock4.0 and Autodock vina [20, 21]. Three TOPK structures were modeled with comparative modeling since TOPK structure has not yet determined by experimental methods. Three predicted structures of the full-length TOPK (left) and their ATP-binding sites (right) are shown in Figure 4A. The results of docking simulation analysis showed that GCM occupied the ATP-binding site of TOPK and fit the binding site very well (Figure 4B & 4C). Specifically, it was found that the terminal alkene of GCM could interact with V46, V98, M115 and V174, which locate at the bottom of ATP-binding site, to form the hydrophobic patch, and the hydroxyl groups of GCM could form the hydrogen bonds with K64, D186 and K121(Figure 4D). These results suggested that GCM was able to bind to the TOPK active site. To experimental validate these results, we performed Sepharose 4B-based an in vitro pull-down assay and the results are shown in Figure 4E. No obvious association was found when the TOPK protein was incubated with Sepharose 4B beads alone, whereas a notably band was observed when TOPK was incubated with GCM–Sepharose 4B beads, supporting the data generated by the docking simulation analysis. To determine whether the interaction between GCM and TOPK caused a direct inactivation of TOPK, we conducted an in vitro kinase assay using histone-H3 protein as a substrate of TOPK. As shown in Figure 4F, exposure to GCM resulted in a dose-dependent reduction of phospho-histone H3 induced by active TOPK. These data clearly supported that TOPK is a direct target of GCM.

### GCM suppresses tumor growth in HepG2 xenograft model

**Figure 5:**
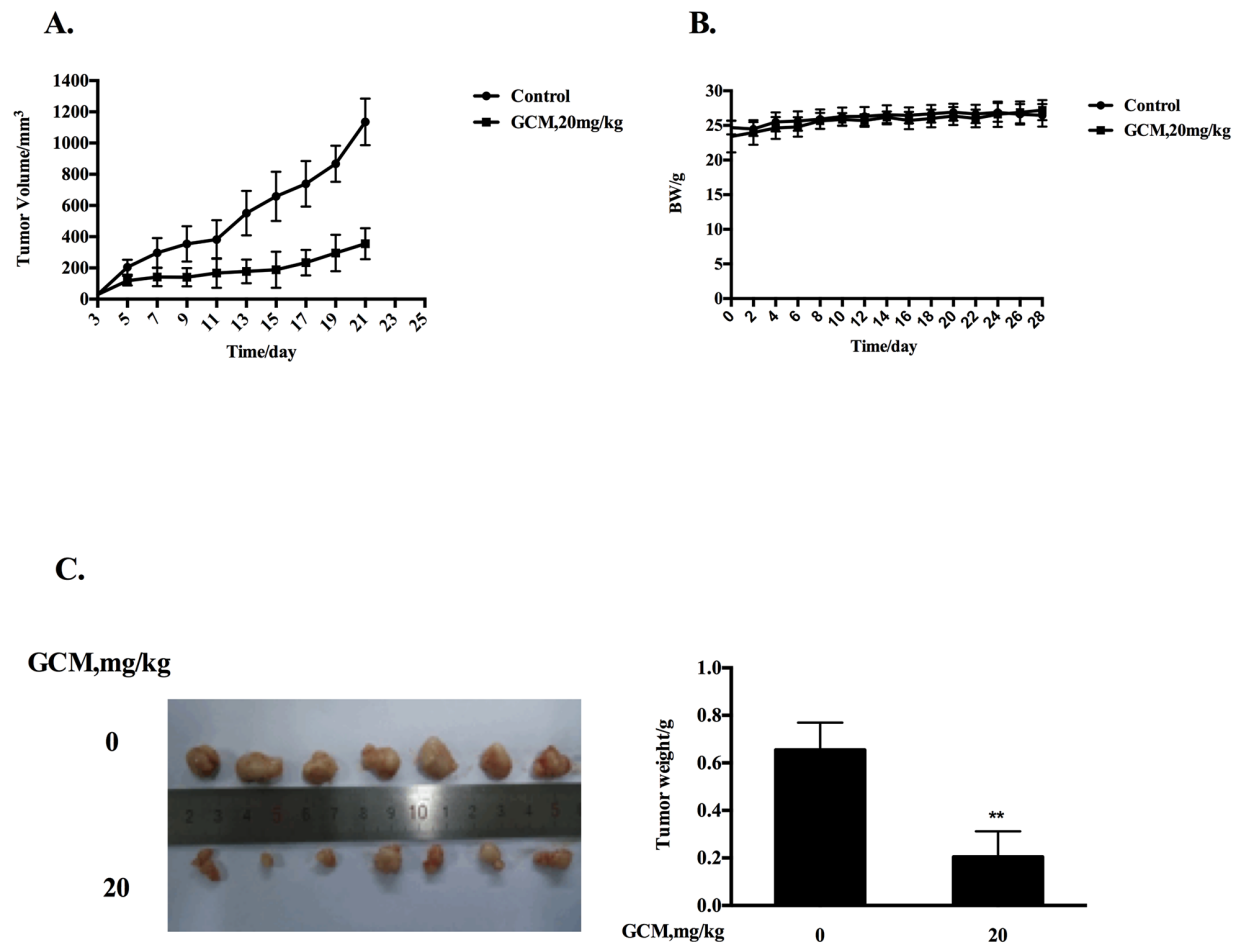
GCM suppresses tumor growth in preventive HepG2 xenograft model GCM was given daily by i.p. injection (20 mg/kg body weight) starting 7 days before s.c. inoculation of HepG2 cells for 28 days. **A.** Inhibitory effects of GCM on tumor growth. **B.** Body weight kinetics of mice **C.** Reduction of final tumor weight.

**Figure 6:**
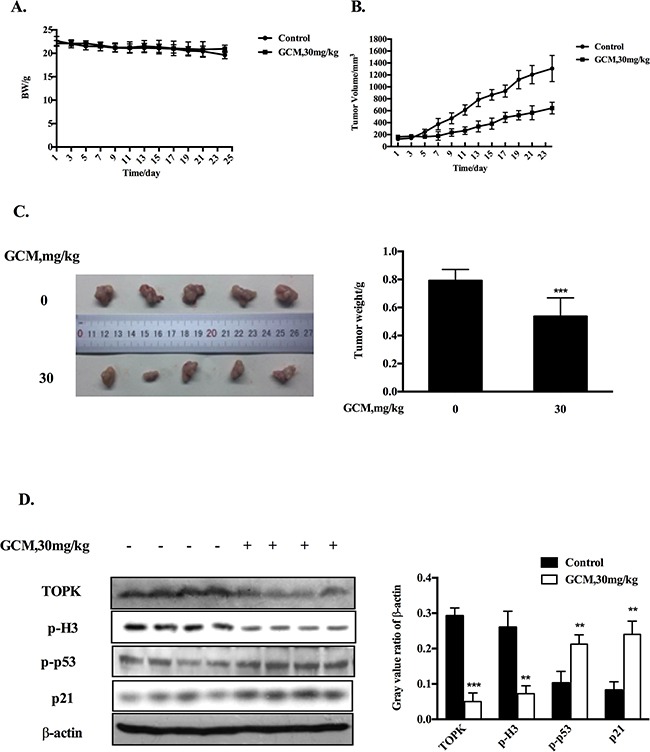
GCM suppresses tumor growth in therapeutic HepG2 xenograft model GCM was given daily by i.p. injection (30 mg/kg body weight) after one week inoculation of HepG2 cells for 24 days. **A.** Body weight kinetics of mice. **B.** Inhibitory effects of GCM on tumor growth. **C.** Reduction of final tumor weight. **D.** Influences of GCM on TOPK activity and p53 signaling assessed by western blotting.

Based on the above results, the key in vitro findings were validated in vivo in a xenograft mouse model of human HepG2 liver cancer. The chemopreventive effect of GCM was first evaluated using the prevention setting, in which GCM was given daily by i.p. injection (20 mg/kg body weight) starting 7 days before s.c. inoculation of HepG2 cells for 28 days. As shown in Figure 5A, exposure to GCM led to a significant suppression of tumor growth (p < 0.01) without affecting body weight of the mice (Figure 5B) and decreased the final tumor weight by 68.8% (Figure 5C, p < 0.05) Having established the chemopreventive efficacy of GCM against liver cancer, we next assessed the therapeutic potential of GCM using the therapeutic setting, in which GCM was given daily by i.p. injection (30 mg/kg body weight) after one week inoculation of HepG2 cells for 24 days. As shown in Figure 6A, the therapeutic dose of GCM (30 mg/kg body weight) did not cause decrease of body weight of the mice. GCM was still effective against the established tumor growth (Figure 6B) with the final tumor weight reduction by 43% (Figure 6C) in comparison with the tumor of the untreated mice. In addition, Mechanistic analysis (Figure 6D) showed that GCM was able to decreased TOPK expression and inhibited its substrate histone H3 phosphorylation. In line with the TOPK inactivation, p53 phosphorylation and its transcriptional target p21 expression were increased compared with that found in untreated tumor tissues, which are consistent with that found in vitro. Together, these data suggested that GCM was capable of suppressing HepG2 liver tumor growth in both prevention and therapeutic settings possibly associated with inactivation of TOPK.

## DISCUSSION

Flavonoids, triterpene saponins, and coumarins have been identified as the main bioactive chemical constituents in licorice [13]. While the investigations on identification of the active anti-cancer constituents from licorice mainly focus on glycyrrhizin [10, 22, 23], a triterpene compound and isoangustone A (IAA) [24–26], a flavonoid compound, little is known about the anti-cancer function of the other components. GCM is a major bioactive coumarin compound isolated from licorice and the anti-cancer activity of GCM has not been scientifically addressed. In the present study, we have tested the anti-liver cancer activity of GCM using both in vitro and in vivo models and found for the first time that GCM possesses a potent activity against liver cancer through mechanisms involved in direct inhibition of TOPK and activation of p53 pathway. Our findings supported GCM as a novel active compound that contributed to the anti-cancer activity of licorice and provide a novel mechanistic support for the anti-cancer function of licorice.

p53 is the first identified and the best known tumor suppressor. Occurrence of p53 mutations is a common feature in HCC [27]. Restoration of p53 is considered to be an attractive approach to cancer treatment. Studies with animal model demonstrated that activation of p53 indeed exhibited a promising therapeutic efficacy against certain types of cancer including HCC [28]. In the present study, our results showed that exposure to GCM led to a dose-dependent p53 activation evidenced by increased p53 phosphorylation, followed by induction of its transcriptional targets p21 and puma. The functional role of p53 activation was determined by using knockdown or knockout approach and the results indicated that the apoptotic and anti-proliferative effect of GCM were significantly attenuated under the condition of p53 deficiency, supporting a critical role of p53 activation in GCM-induced apoptosis and cell growth inhibition.

Having established the role of p53 in the anti-cancer activity of GCM, we next investigated the mechanisms of p53 activation in response to GCM. TOPK is an oncogenic kinase that has been found overexpressed in many types of cancer including HCC [7]. A number of mechanisms have been postulated to contribute to oncogenic activity of TOPK. One of them was its ability to bind to and inactivate p53 protein [19]. We therefore questioned whether GCM-triggered p53 activation was due to inactivation of TOPK. Our data demonstrated that GCM was able to decrease both phospho- and total TOPK in HepG2 cells. These effects were also observed in several other cancer cell lines, suggesting its general application. Moreover, inhibition of TOPK by its siRNA indeed caused p53 transcriptional activation and cell number reduction in HepG2 cells, supporting the role of TOPK inhibition in GCM-induced p53 activation and TOPK could be an effective target for HCC chemoprevention and therapy. Regarding the mechanism of TOPK inhibition by GCM, our data showed that GCM could bind to TOPK evidenced by the docking simulation and pull-down assay, and inhibit GCM kinase activity (Figure 4). Our data also indicated that GCM was able to promote TOPK degradation (Figure 3B). We speculated that the binding of GCM and TOPK affected phosphorylation status of TOPK and the changes of TOPK phosphorylation may influence its stability. Further experimental validation of this notion is clearly needed.

Finally, we translated our cell culture findings into HepG2 xenograft model. The results indicated that p53 was indeed activated by GCM treatment in vivo evidenced by increased p53 phosphorylation and up-regulation of its transcriptional target p21. Consistent with p53 activation, TOPK was inhibited in vivo in response to GCM evidenced by decreased TOPK protein level and its substrate histone H3 phosphorylation (Figure 6D). Moreover, in both preventive and therapeutic settings, a significantly inhibitory effect on tumor growth by GCM was observed (Figure 5A&5B, Figure 6A&6B). Our findings provided the first evidence that GCM was able to inhibit TOPK, activate p53/p21 axis and suppress tumor growth in vivo.

In summary, GCM was capable of directly inactivating oncogenic kinase TOPK, which in turn led to activation of its binding partner p53, followed by cell cycle arrest and cell death induction in vitro and tumor reduction in vivo. Our findings supported TOPK could be a potential target for HCC treatment and GCM holds great potential as a novel HCC chemopreventive and therapeutic agent.

## MATERIALS AND METHODS

### Chemicals and reagents

GCM (purity 98%) was isolated from the licorice (Glycyrrhiza uralensis) by the authors as reported previously [13]. Antibodies specific for caspase−3, −8, c-parp, c-caspase-3, c-caspase-9, total TOPK, phospho-TOPK, phospho-histone H3, puma, p21 and phospho-p53 were purchased from Cell Signaling Technology (Beverly, MA). siRNAs for p53 or TOPK and nontargeting siRNA were purchased from Life Technologies, Inc. (Life Technologies, MD). Antibody for β-actin was purchased from MBL International Corporation.

### Cell culture and treatments

Human hepatocellular carcinoma HepG2, Huh7 and human prostate cancer DU-145 cells were grown in Dulbecco's Modification of Eagle's Medium (DMEM) supplemented with 10% fetal bovine serum without antibiotics. Human colon cancer HCT-116 cells were cultured in McCoy's 5 A medium containing 10% fetal bovine serum without antibiotics. At 24-48 h after plating when Cells were 50-60% confluence, the medium was changed before starting the treatment with GCM and/or other agents.

### Apoptosis evaluation

Apoptosis was assessed by Annexin V staining of externalized phosphatidylserine in apoptotic cells by flow cytometry using Annexin V/FITC Staining Kit from MBL International (Woburn, MA).

### Western blotting

The cell lysate was prepared in ice-cold radioimmuno- precipitation assay buffer. Cell lysate proteins were separated by electrophoresis and transferred to a nitrocellulose membrane (Millipore). The blot was then probed with a primary antibody followed by incubation with the appropriate horseradish peroxidase-conjugated secondary antibodies. The immunoreactive bands were visualized by enhanced chemiluminescence (Fisher/Pierce) and recorded on an X-ray film.

### Cell cycle analysis

Cell cycle distribution was measured by flow cytometry analysis of DNA content following staining with propidium iodide (PI).

### RNA interference

HepG2 cells were transfected with 5 nmol/L of TOPK siRNA or non-targeting siRNA using INTERFER siRNA transfection reagent according to the manufacturer's instructions (Polyplus-Transfection, Inc., New York, NY) for 36 h and then were used for subsequent experiments.

### Molecular modeling of TOPK and GCM

The TOPK structure was not solved by experimental methods so far, and therefore was predicted in silico in this study. The sequence of TOPK was downloaded from National Center for Biotechnology Information (Gene Bank: Q96KB5). Three well-known and popular web servers, including RaptorX from Xu lab [29], I-TASSER from Zhang lab [30] and Robetta from Baker lab [31], which provide the service of the protein structure-prediction, were used to build TOPK structure by using comparative modeling methods. The protein structure from 4YU9, 2NRU, and 4W7P (PDB entry) were selected as the template structure by these three web servers, respectively. Three predicted TOPK structures present the similar conformational topology of protein kinase. Especially, the protein backbone RMSD for the small-molecule binding site of these three predicted TOPK structures is 2.5-3.5 Å. Subsequently, GCM was docked into these three predicted TOPK structures with both Autodock4.0 and Autodock vina [20, 21], and the docking parameters were kept as default. Based on the predicted interaction energies and interaction mode between GCM and protein, all docking results were summarized and analyzed, and a most probable interaction model was concluded.

### In vitro pull-down assay

HepG2 cell lysates (1 mg) were incubated with GCM-Sepharose 4B or Sepharose 4B alone in the reaction buffer [50 mM Tris (pH 7.5), 5 mM ethylenediaminetetraacetic acid (EDTA), 150 mM NaCl, 1 mM dithiothreitol (DTT), 0.01% Nonidet P-40, 2 μg/ml bovine serum albumin, 0.02 mM phenylmethylsulfonyl fluoride (PMSF) and 1 μg/ml protease inhibitor mixture] at 4°C overnight. The beads were washed five times and then beads were used for western blotting analysis.

### In vitro kinase assay

Inactive histone H3 proteins (1 μg) were used as the substrate for an in vitro kinase assay with 1.5 μg of active TOPK. Reactions were performed in 1×kinase buffer (25 mM Tris (pH 7.5), 5 mM b-glycerophosphate, 2 mM DTT, 0.1mM Na3VO4, 10 mM MgCl_2_, and 5 mM MnCl_2_) with 100 μM ATP at 32°C for 1.5 h. Reactions were stopped by adding 5×SDS sample buffer. Phospho, total histone H3 and TOPK were analyzed by western blotting.

### Animals and treatments

The anti-cancer activity of GCM was evaluated by both prevention and therapeutic settings. Animal Care and procedures were approved by the Institutional Animal Care and Use Committee. To establish the cancer xenograft, 2×106 HepG2 cells were mixed with Matrigel (50%) (Becton Dickinson) and injected subcutaneous (s.c.) into the right flank of 7- to 8-wk-old male BALB/c athymic nude mice (Charles River Laboratories). For the prevention setting, GCM was given daily by i.p. injection (20 mg/kg body weight) starting 7 days before s.c. inoculation of HepG2 cells for 28 days. For the therapeutic setting, GCM (30mg per kg body weight) was given daily by i.p. injection (30 mg/kg body weight) when the average tumor volume reached about 100 mm3 (after about one week inoculation) for 24 days. Tumors were measured with a caliper and tumor volumes were calculated using the following formula: 1/2 (w_1_*w_2_*w_2_), where w1 is the largest tumor diameter and w2 is the smallest tumor diameter. A portion of the tumors from control and treated animals was used for preparation of tumor lysate used in further analysis.

### Statistical analysis

Data are presented as mean ±SD. These data were analyzed with the ANOVA with appropriate post-hoc comparison among means. p<0.05 was considered statistically significant.
